# Dicyanido[tris­(2-pyridyl­meth­yl)amine]­cobalt(III) hexa­fluorido­phosphate

**DOI:** 10.1107/S1600536811012001

**Published:** 2011-04-13

**Authors:** Fan Yu, Bao Li

**Affiliations:** a School of Chemistry and Environmental Engineering, Jianghan University, Wuhan, Hubei 430056, People’s Republic of China; b Department of Chemistry and Chemical Engineering, Huazhong University of Science and Technology, Wuhan 430074, People’s Republic of China

## Abstract

In the title complex, [Co(CN)_2_(C_18_H_18_N_4_)]PF_6_, the Co^III^ atom together with one of the pyridyl rings and two cyanide anions are located on a mirror plane, while the P atom is located on an inversion centre. The Co^III^ atom exhibits an octa­hedral geometry, coordinated by four N atoms from the tris­(2-pyridyl­meth­yl)amine ligand with an average Co—N distance of 1.953 (2) Å, and two cyanide C atoms with an average Co—C distance of 1.895 (2) Å. The crystal packing is stabilized by inter­molecular C—H⋯N and C—H⋯F inter­actions.

## Related literature

For related structures, see: Guo *et al.* (2007 ▶), Liu *et al.* (2010 ▶).
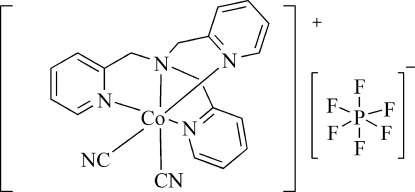


## Experimental

### 

#### Crystal data

[Co(CN)_2_(C_18_H_18_N_4_)]PF_6_
*M* *_r_* = 546.30
Orthorhombic, 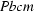

*a* = 10.703 (2) Å
*b* = 13.472 (3) Å
*c* = 15.151 (3) Å
*V* = 2184.7 (8) Å^3^
*Z* = 4
Mo *K*α radiation
μ = 0.93 mm^−1^
*T* = 293 K
0.40 × 0.30 × 0.25 mm

#### Data collection

Bruker SMART APEX diffractometer
Absorption correction: multi-scan (*SADABS*; Sheldrick, 1996 ▶) *T*_min_ = 0.722, *T*_max_ = 0.792
26914 measured reflections
2173 independent reflections
2053 reflections with *I* > 2σ(*I*)
*R*_int_ = 0.028

#### Refinement

*R*[*F* ^2^ > 2σ(*F* ^2^)] = 0.025
*wR*(*F* ^2^) = 0.070
*S* = 1.08
2173 reflections
176 parameters
H-atom parameters constrained
Δρ_max_ = 0.27 e Å^−3^
Δρ_min_ = −0.24 e Å^−3^

### 

Data collection: *SMART* (Bruker, 1997 ▶); cell refinement:  *SAINT* (Bruker, 1999 ▶); data reduction: *SAINT*; program(s) used to solve structure: *SHELXS97* (Sheldrick, 2008 ▶); program(s) used to refine structure: *SHELXL97* (Sheldrick, 2008 ▶); molecular graphics: *SHELXTL* (Sheldrick, 2008 ▶); software used to prepare material for publication: *SHELXTL* (Sheldrick, 2008 ▶).

## Supplementary Material

Crystal structure: contains datablocks I, global. DOI: 10.1107/S1600536811012001/vm2083sup1.cif

Structure factors: contains datablocks I. DOI: 10.1107/S1600536811012001/vm2083Isup2.hkl

Additional supplementary materials:  crystallographic information; 3D view; checkCIF report

## Figures and Tables

**Table 1:** Hydrogen-bond geometry (Å, °)

*D*—H⋯*A*	*D*—H	H⋯*A*	*D*⋯*A*	*D*—H⋯*A*
C3—H3*A*⋯N4^i^	0.93	2.60	3.339 (3)	137
C6—H6*A*⋯F3^i^	0.96	2.29	3.234 (2)	169
C7—H7*A*⋯F2^ii^	0.93	2.44	3.128 (3)	131
C9—H9*A*⋯N5^iii^	0.93	2.57	3.410 (2)	151

Symmetry codes: (i) 
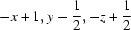
; (ii) 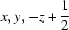; (iii) 
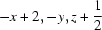
.

**Fig. 1.**
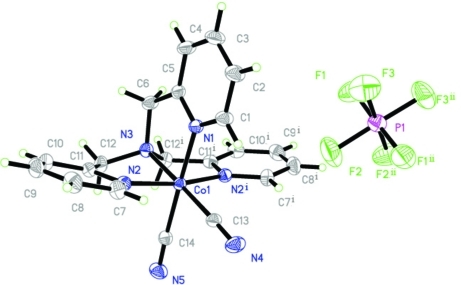
Molecular structure of the title compound showing atomic numbering and 30% probability displacement ellipsoids.Symmetry codes: (i) x,y,-z + 1/2; (ii) x,-y + 1/2,-z.

**Fig. 2.**
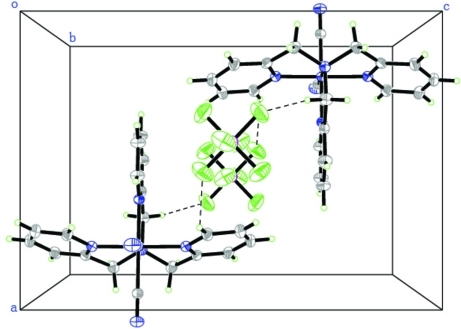
The packing of title compound, viewed down the b axis. Symmetry codes: (i) x,y,-z + 1/2; (ii) x,-y + 1/2,-z.

## supplementary crystallographic information

### Comment

Transitional metal-cyanide systems have been extensively investigated due to their versatile structure and physical properties, especially in the field of molecular-based magnets. A large number of cyanide-bridged heterobimetallic or homometallic coordination complexes that exhibit excellent magnetic properties has been constructed by using the hexacyanoferrate(III) and hexacyanocobaltate(III) anions as templates (Liu *et al.*, 2010). In constrast, complexes constructed by using the octacyanotungsten(IV) anion are rare. In an attempt to synthesize new complexes, we decided to use octacyanotungsten(IV) anions as template and new compounds containing cyanides have been obtained. The octacyanotungsten(IV) anion was not coordinated to Co atom *via* cyanide bridges, but acts as source of *in situ* cyanide generation.

In the title compound, the cobalt(III) centers are coordinated by two cyanides and tris(2-pyridylmethyl)amine (Fig. 1). Each cobalt(III) ion is coordinated by four N atoms with average Co—N distance of 1.957 (2) Å and two C atoms with average Co—C distance of 1.895 (2) Å in a rigid octahedral geometry, in accordance with those observed in other [Co(N)_4_(CN)_2_]^-^ units (Guo *et al.*, 2007). The dihedral angle of two types of pyridyl rings is about 80.17°, indicating the nearly perpendicular occupation of these pyridyl rings. The crystal packing is stabilized by C—H···N and C—H···F hydrogen bonding interactons (Table 1, Fig. 2).

### Experimental

0.01 mmol K_4_W(CN)_8_ in 5 ml H_2_O was added in a tube, and 3 ml H_2_O was layered on as a buffer. A solution containing 0.1 mmol CoCl_2_.6H_2_O, 0.11 mmol tris(2-pyridylmethyl)amine and 0.11 mmol KPF_6_ in 5 mL acetone and 1 ml H_2_O was stirred for 30 min and layered on top of the previous solution. After half a year, yellow crystals were obtained.

### Refinement

All H atoms were placed geometrically with C—H = 0.93 (aromatic) or 0.96-0.97 Å (CH_2_) , and refined using a riding atom model with their isotropic displacement factors, *U*_ĩso_ fixed at 1.2 time the *U*_eq_ of the parent C atom.

### Crystal data

**Table:**

[Co(CN)_2_(C_18_H_18_N_4_)]PF_6_	*F*(000) = 1104
*M**_r_* = 546.30	*D*_x_ = 1.658 Mg m^−^^3^*D*_m_ = 1.658 Mg m^−^^3^*D*_m_ measured by not measured
Orthorhombic, *P**b**c**m*	Mo *K*α radiation, λ = 0.71073 Å
Hall symbol: -P 2c 2b	Cell parameters from 27644 reflections
*a* = 10.703 (2) Å	θ = 6.1–54.9°
*b* = 13.472 (3) Å	µ = 0.93 mm^−^^1^
*c* = 15.151 (3) Å	*T* = 293 K
*V* = 2184.7 (8) Å^3^	Block, yellow
*Z* = 4	0.40 × 0.30 × 0.25 mm

### Data collection

**Table:**

Bruker SMART APEX diffractometer	2173 independent reflections
Radiation source: fine-focus sealed tube	2053 reflections with *I* > 2σ(*I*)
graphite	*R*_int_ = 0.028
Detector resolution: 0 pixels mm^-1^	θ_max_ = 26.0°, θ_min_ = 3.3°
ω scans	*h* = −13→13
Absorption correction: multi-scan (*SADABS*; Sheldrick, 1996)	*k* = −16→14
*T*_min_ = 0.722, *T*_max_ = 0.792	*l* = −18→18
26914 measured reflections	

### Refinement

**Table:**

Refinement on *F*^2^	Primary atom site location: structure-invariant direct methods
Least-squares matrix: full	Secondary atom site location: difference Fourier map
*R*[*F*^2^ > 2σ(*F*^2^)] = 0.025	Hydrogen site location: inferred from neighbouring sites
*wR*(*F*^2^) = 0.070	H-atom parameters constrained
*S* = 1.08	*w* = 1/[σ^2^(*F*_o_^2^) + (0.043*P*)^2^ + 0.5717*P*] where *P* = (*F*_o_^2^ + 2*F*_c_^2^)/3
2173 reflections	(Δ/σ)_max_ < 0.001
176 parameters	Δρ_max_ = 0.27 e Å^−^^3^
0 restraints	Δρ_min_ = −0.24 e Å^−^^3^
1.234 constraints	

### Special details

**Table:**

Geometry. All esds (except the esd in the dihedral angle between two l.s. planes) are estimated using the full covariance matrix. The cell esds are taken into account individually in the estimation of esds in distances, angles and torsion angles; correlations between esds in cell parameters are only used when they are defined by crystal symmetry. An approximate (isotropic) treatment of cell esds is used for estimating esds involving l.s. planes.
Refinement. Refinement of *F*^2^ against ALL reflections. The weighted *R*-factor *wR* and goodness of fit *S* are based on *F*^2^, conventional *R*-factors *R* are based on *F*, with *F* set to zero for negative *F*^2^. The threshold expression of *F*^2^ > σ(*F*^2^) is used only for calculating *R*-factors(gt) *etc*. and is not relevant to the choice of reflections for refinement. *R*-factors based on *F*^2^ are statistically about twice as large as those based on *F*, and *R*- factors based on ALL data will be even larger.

### Fractional atomic coordinates and isotropic or equivalent isotropic displacement parameters (Å^2^)

**Table:**

	*x*	*y*	*z*	*U*_iso_*/*U*_eq_	
Co1	0.82856 (2)	0.057628 (17)	0.2500	0.02656 (10)	
N1	0.64937 (15)	0.02901 (13)	0.2500	0.0308 (3)	
N2	0.82831 (10)	0.04135 (9)	0.37718 (8)	0.0326 (3)	
N3	0.85307 (16)	−0.08775 (13)	0.2500	0.0328 (4)	
N4	0.8016 (2)	0.28241 (14)	0.2500	0.0499 (5)	
N5	1.10402 (19)	0.11315 (17)	0.2500	0.0508 (5)	
C1	0.5562 (2)	0.09626 (16)	0.2500	0.0412 (5)	
H1A	0.5761	0.1634	0.2500	0.049*	
C2	0.4321 (2)	0.0686 (2)	0.2500	0.0501 (6)	
H2A	0.3694	0.1163	0.2500	0.060*	
C3	0.4029 (2)	−0.0304 (2)	0.2500	0.0503 (6)	
H3A	0.3199	−0.0507	0.2500	0.060*	
C4	0.4976 (2)	−0.09968 (17)	0.2500	0.0470 (5)	
H4A	0.4792	−0.1671	0.2500	0.056*	
P1	0.43435 (6)	0.2500	0.0000	0.04841 (17)	
C6	0.7279 (2)	−0.13885 (15)	0.2500	0.0408 (5)	
H6A	0.7219	−0.1806	0.3012	0.049*	
F2	0.54077 (17)	0.23947 (14)	0.07289 (13)	0.1109 (6)	
F1	0.43287 (15)	0.13526 (10)	−0.01878 (13)	0.0969 (5)	
C13	0.80941 (18)	0.19751 (15)	0.2500	0.0333 (4)	
C8	0.76782 (18)	0.07851 (15)	0.52469 (11)	0.0524 (4)	
H8A	0.7322	0.1225	0.5647	0.063*	
F3	0.32765 (16)	0.23608 (13)	0.07209 (10)	0.0978 (5)	
C10	0.86385 (17)	−0.07628 (14)	0.49189 (11)	0.0447 (4)	
H10A	0.8939	−0.1378	0.5098	0.054*	
C7	0.77754 (15)	0.10373 (12)	0.43633 (10)	0.0417 (3)	
H7A	0.7483	0.1652	0.4175	0.050*	
C12	0.92406 (15)	−0.11166 (11)	0.33220 (10)	0.0400 (3)	
H12A	1.0122	−0.0977	0.3241	0.048*	
H12B	0.9146	−0.1813	0.3469	0.048*	
C9	0.81121 (17)	−0.01197 (16)	0.55256 (11)	0.0510 (4)	
H9A	0.8053	−0.0299	0.6117	0.061*	
C14	1.0012 (2)	0.08893 (15)	0.2500	0.0340 (4)	
C11	0.87139 (14)	−0.04806 (10)	0.40424 (10)	0.0357 (3)	
C5	0.6211 (2)	−0.06763 (14)	0.2500	0.0336 (4)	

### Atomic displacement parameters (Å^2^)

**Table:**

	*U*^11^	*U*^22^	*U*^33^	*U*^12^	*U*^13^	*U*^23^
Co1	0.02638 (16)	0.02409 (15)	0.02921 (15)	0.00029 (9)	0.000	0.000
N1	0.0296 (8)	0.0301 (8)	0.0326 (8)	−0.0016 (6)	0.000	0.000
N2	0.0312 (6)	0.0339 (6)	0.0328 (6)	−0.0008 (4)	−0.0014 (4)	−0.0005 (5)
N3	0.0355 (9)	0.0281 (8)	0.0348 (8)	0.0041 (7)	0.000	0.000
N4	0.0456 (11)	0.0296 (9)	0.0746 (14)	−0.0006 (8)	0.000	0.000
N5	0.0318 (10)	0.0664 (13)	0.0541 (11)	−0.0040 (9)	0.000	0.000
C1	0.0311 (10)	0.0343 (10)	0.0583 (13)	0.0002 (8)	0.000	0.000
C2	0.0318 (12)	0.0533 (14)	0.0654 (16)	0.0017 (9)	0.000	0.000
C3	0.0301 (11)	0.0612 (14)	0.0595 (14)	−0.0113 (10)	0.000	0.000
C4	0.0449 (13)	0.0394 (11)	0.0566 (13)	−0.0155 (10)	0.000	0.000
P1	0.0461 (3)	0.0473 (3)	0.0519 (3)	0.000	0.000	−0.0053 (3)
C6	0.0452 (12)	0.0269 (9)	0.0505 (12)	−0.0053 (8)	0.000	0.000
F2	0.0979 (12)	0.1154 (13)	0.1194 (13)	0.0305 (10)	−0.0483 (10)	0.0004 (10)
F1	0.0994 (11)	0.0502 (7)	0.1411 (14)	−0.0045 (7)	0.0406 (10)	−0.0180 (8)
C13	0.0278 (9)	0.0317 (10)	0.0403 (10)	−0.0021 (7)	0.000	0.000
C8	0.0529 (10)	0.0681 (11)	0.0362 (8)	0.0019 (9)	0.0042 (7)	−0.0091 (8)
F3	0.0994 (12)	0.1130 (13)	0.0809 (10)	−0.0082 (9)	0.0396 (8)	−0.0240 (9)
C10	0.0443 (8)	0.0509 (9)	0.0389 (8)	−0.0040 (7)	−0.0058 (7)	0.0106 (7)
C7	0.0447 (9)	0.0440 (8)	0.0363 (7)	0.0040 (6)	0.0014 (6)	−0.0046 (6)
C12	0.0448 (8)	0.0345 (7)	0.0409 (8)	0.0085 (6)	−0.0051 (6)	0.0058 (6)
C9	0.0497 (9)	0.0721 (12)	0.0314 (7)	−0.0062 (8)	0.0000 (7)	0.0057 (8)
C14	0.0330 (11)	0.0348 (9)	0.0341 (9)	0.0025 (8)	0.000	0.000
C11	0.0330 (7)	0.0379 (7)	0.0361 (7)	−0.0021 (6)	−0.0041 (6)	0.0044 (6)
C5	0.0383 (11)	0.0328 (10)	0.0296 (9)	−0.0057 (8)	0.000	0.000

### Geometric parameters (Å, °)

**Table:**

Co1—C14	1.895 (2)	C4—C5	1.390 (3)
Co1—C13	1.896 (2)	C4—H4A	0.9300
Co1—N2^i^	1.9393 (13)	P1—F1^ii^	1.5719 (14)
Co1—N2	1.9393 (13)	P1—F1	1.5719 (14)
Co1—N1	1.9563 (17)	P1—F3^ii^	1.5913 (15)
Co1—N3	1.9760 (18)	P1—F3	1.5913 (15)
N1—C5	1.337 (3)	P1—F2	1.5928 (16)
N1—C1	1.347 (3)	P1—F2^ii^	1.5928 (16)
N2—C7	1.343 (2)	C6—C5	1.492 (3)
N2—C11	1.3534 (19)	C6—H6A	0.9600
N3—C12	1.4940 (17)	C8—C9	1.371 (3)
N3—C12^i^	1.4940 (17)	C8—C7	1.385 (2)
N3—C6	1.507 (3)	C8—H8A	0.9300
N4—C13	1.147 (3)	C10—C9	1.383 (3)
N5—C14	1.148 (3)	C10—C11	1.384 (2)
C1—C2	1.379 (3)	C10—H10A	0.9300
C1—H1A	0.9300	C7—H7A	0.9300
C2—C3	1.370 (4)	C12—C11	1.498 (2)
C2—H2A	0.9300	C12—H12A	0.9700
C3—C4	1.378 (4)	C12—H12B	0.9700
C3—H3A	0.9300	C9—H9A	0.9300
			
C14—Co1—C13	83.35 (8)	F1^ii^—P1—F3	89.11 (10)
C14—Co1—N2^i^	91.52 (3)	F1—P1—F3	90.06 (9)
C13—Co1—N2^i^	96.45 (4)	F3^ii^—P1—F3	88.28 (14)
C14—Co1—N2	91.52 (3)	F1^ii^—P1—F2	88.24 (9)
C13—Co1—N2	96.45 (4)	F1—P1—F2	92.58 (10)
N2^i^—Co1—N2	167.01 (7)	F3^ii^—P1—F2	178.30 (9)
C14—Co1—N1	178.51 (8)	F3—P1—F2	91.54 (11)
C13—Co1—N1	95.16 (8)	F1^ii^—P1—F2^ii^	92.58 (10)
N2^i^—Co1—N1	88.64 (3)	F1—P1—F2^ii^	88.24 (9)
N2—Co1—N1	88.64 (3)	F3^ii^—P1—F2^ii^	91.54 (11)
C14—Co1—N3	95.23 (8)	F3—P1—F2^ii^	178.30 (9)
C13—Co1—N3	178.58 (8)	F2—P1—F2^ii^	88.69 (16)
N2^i^—Co1—N3	83.58 (4)	C5—C6—N3	112.79 (17)
N2—Co1—N3	83.58 (4)	C5—C6—H6A	109.0
N1—Co1—N3	86.26 (7)	N3—C6—H6A	109.0
C5—N1—C1	119.17 (18)	N4—C13—Co1	177.95 (19)
C5—N1—Co1	114.45 (14)	C9—C8—C7	119.36 (16)
C1—N1—Co1	126.38 (15)	C9—C8—H8A	120.3
C7—N2—C11	119.50 (13)	C7—C8—H8A	120.3
C7—N2—Co1	126.35 (11)	C9—C10—C11	119.30 (16)
C11—N2—Co1	113.65 (10)	C9—C10—H10A	120.3
C12—N3—C12^i^	112.94 (16)	C11—C10—H10A	120.3
C12—N3—C6	110.72 (10)	N2—C7—C8	121.45 (16)
C12^i^—N3—C6	110.72 (10)	N2—C7—H7A	119.3
C12—N3—Co1	106.33 (9)	C8—C7—H7A	119.3
C12^i^—N3—Co1	106.33 (9)	N3—C12—C11	107.02 (12)
C6—N3—Co1	109.56 (12)	N3—C12—H12A	110.3
N1—C1—C2	122.0 (2)	C11—C12—H12A	110.3
N1—C1—H1A	119.0	N3—C12—H12B	110.3
C2—C1—H1A	119.0	C11—C12—H12B	110.3
C3—C2—C1	118.9 (2)	H12A—C12—H12B	108.6
C3—C2—H2A	120.6	C8—C9—C10	119.36 (15)
C1—C2—H2A	120.6	C8—C9—H9A	120.3
C2—C3—C4	119.4 (2)	C10—C9—H9A	120.3
C2—C3—H3A	120.3	N5—C14—Co1	176.3 (2)
C4—C3—H3A	120.3	N2—C11—C10	121.03 (15)
C3—C4—C5	119.3 (2)	N2—C11—C12	114.62 (13)
C3—C4—H4A	120.4	C10—C11—C12	124.34 (14)
C5—C4—H4A	120.4	N1—C5—C4	121.2 (2)
F1^ii^—P1—F1	178.85 (13)	N1—C5—C6	116.95 (18)
F1^ii^—P1—F3^ii^	90.06 (9)	C4—C5—C6	121.88 (19)
F1—P1—F3^ii^	89.11 (10)		
			
C14—Co1—N1—C5	180.0	C2—C3—C4—C5	0.0
C13—Co1—N1—C5	180.0	C12—N3—C6—C5	116.97 (11)
N2^i^—Co1—N1—C5	83.65 (4)	C12^i^—N3—C6—C5	−116.97 (11)
N2—Co1—N1—C5	−83.65 (4)	Co1—N3—C6—C5	0.0
N3—Co1—N1—C5	0.0	C14—Co1—C13—N4	0.0
C14—Co1—N1—C1	0.0	N2^i^—Co1—C13—N4	−90.79 (3)
C13—Co1—N1—C1	0.0	N2—Co1—C13—N4	90.79 (3)
N2^i^—Co1—N1—C1	−96.35 (4)	N1—Co1—C13—N4	180.0
N2—Co1—N1—C1	96.35 (4)	N3—Co1—C13—N4	0.0
N3—Co1—N1—C1	180.0	C11—N2—C7—C8	−0.2 (2)
C14—Co1—N2—C7	107.30 (14)	Co1—N2—C7—C8	171.16 (13)
C13—Co1—N2—C7	23.82 (14)	C9—C8—C7—N2	0.2 (3)
N2^i^—Co1—N2—C7	−149.2 (2)	C12^i^—N3—C12—C11	156.60 (11)
N1—Co1—N2—C7	−71.22 (14)	C6—N3—C12—C11	−78.58 (16)
N3—Co1—N2—C7	−157.61 (14)	Co1—N3—C12—C11	40.35 (14)
C14—Co1—N2—C11	−80.90 (11)	C7—C8—C9—C10	0.0 (3)
C13—Co1—N2—C11	−164.38 (11)	C11—C10—C9—C8	−0.1 (3)
N2^i^—Co1—N2—C11	22.6 (4)	C13—Co1—C14—N5	0.0
N1—Co1—N2—C11	100.58 (11)	N2^i^—Co1—C14—N5	96.31 (4)
N3—Co1—N2—C11	14.19 (11)	N2—Co1—C14—N5	−96.31 (4)
C14—Co1—N3—C12	60.30 (10)	N1—Co1—C14—N5	0.0
C13—Co1—N3—C12	60.30 (10)	N3—Co1—C14—N5	180.0
N2^i^—Co1—N3—C12	151.25 (11)	C7—N2—C11—C10	0.1 (2)
N2—Co1—N3—C12	−30.65 (10)	Co1—N2—C11—C10	−172.35 (12)
N1—Co1—N3—C12	−119.70 (10)	C7—N2—C11—C12	179.11 (14)
C14—Co1—N3—C12^i^	−60.30 (10)	Co1—N2—C11—C12	6.70 (16)
C13—Co1—N3—C12^i^	−60.30 (10)	C9—C10—C11—N2	0.1 (2)
N2^i^—Co1—N3—C12^i^	30.65 (10)	C9—C10—C11—C12	−178.84 (16)
N2—Co1—N3—C12^i^	−151.25 (11)	N3—C12—C11—N2	−31.69 (18)
N1—Co1—N3—C12^i^	119.70 (10)	N3—C12—C11—C10	147.32 (16)
C14—Co1—N3—C6	180.0	C1—N1—C5—C4	0.0
C13—Co1—N3—C6	180.0	Co1—N1—C5—C4	180.0
N2^i^—Co1—N3—C6	−89.05 (3)	C1—N1—C5—C6	180.0
N2—Co1—N3—C6	89.05 (3)	Co1—N1—C5—C6	0.0
N1—Co1—N3—C6	0.0	C3—C4—C5—N1	0.0
C5—N1—C1—C2	0.0	C3—C4—C5—C6	180.0
Co1—N1—C1—C2	180.0	N3—C6—C5—N1	0.0
N1—C1—C2—C3	0.0	N3—C6—C5—C4	180.0
C1—C2—C3—C4	0.0		

Symmetry codes: (i) *x*, *y*, −*z*+1/2; (ii) *x*, −*y*+1/2, −*z*.

### Hydrogen-bond geometry (Å, °)

**Table:**

*D*—H···*A*	*D*—H	H···*A*	*D*···*A*	*D*—H···*A*
C3—H3A···N4^iii^	0.93	2.60	3.339 (3)	137
C6—H6A···F3^iii^	0.96	2.29	3.234 (2)	169
C7—H7A···F2^i^	0.93	2.44	3.128 (3)	131
C9—H9A···N5^iv^	0.93	2.57	3.410 (2)	151

Symmetry codes: (iii) −*x*+1, *y*−1/2, −*z*+1/2; (i) *x*, *y*, −*z*+1/2; (iv) −*x*+2, −*y*, *z*+1/2.
